# Turán type inequalities for generalized Mittag-Leffler function

**DOI:** 10.1186/s13660-017-1475-z

**Published:** 2017-08-29

**Authors:** Xiang Kai Dou, Li Yin

**Affiliations:** Department of Mathematics, Binzhou University, Binzhou City, Shandong Province 256603 China

**Keywords:** 33E12, 26D07, Turán type inequalities, generalized Mittag-Leffler function, $(p,k)$-gamma function

## Abstract

In this paper, we show several Turán type inequalities for a generalized Mittag-Leffler function with four parameters via the $(p,k)$-gamma function.

## Introduction and main results

In 1950, Turán established a remarkable inequality in the special function theory,

$$\bigl[ {P_{n + 1} (r)} \bigr]^{2} > P_{n} (r)P_{n + 2} (r) $$

for all $r\in(-1,1)$ and $n\in\mathbf{N}$, where $P_{n}$ is the Legendre polynomial, that is,

$$P_{n} (r) = F \biggl( { - n,n + 1;1;\frac{{1 - r}}{2}} \biggr). $$

Here, for given complex numbers *a*, *b* and *c* with $c\neq 0,-1,-2,\ldots$ , the *Gaussian hypergeometric function* is the analytic continuation to the slit place $\mathbb{C}\setminus[1,\infty )$ of the series

$$F(a,b;c;z)={}_{2}F_{1}(a,b;c;z)=\sum ^{\infty}_{n=0}\frac{(a,n)(b,n)}{ (c,n)}\frac{z^{n}}{n!}, \quad|z|< 1. $$

Here, $(a,0)=1$ for $a\neq0$, and $(a,n)$ is the *shifted factorial function* or the *Appell symbol*

$$(a,n)=a(a+1) (a+2)\cdots(a+n-1) $$

for $n\in\mathbb{Z}_{+}$; see [1, 2]. There is an extensive topic dealing with Turán type inequalities, and it has been generalized in many directions for various orthogonal, polynomial and special functions.

The Mittag-Leffler function is defined by

1.1 $$ E_{\alpha,\beta} (z) = \sum_{n = 0}^{\infty}{ \frac{{z^{n} }}{{\Gamma ( {\alpha n + \beta} )}},\quad z,\alpha,\beta\in\mathbb {C},{\operatorname{Re} } } (\alpha) > 0,{\operatorname{Re} } (\beta) > 0, $$

where $\Gamma( \cdot)$ is a classical gamma function. The Mittag-Leffler function plays an important role in several branches of mathematics and engineering sciences, such as statistics, chemistry, mechanics, quantum physics, informatics and others. In particular, it is involved in the explicit formula for the resolvent of Riemann-Liouville fractional integrals by Hille and Tamarkin. Many properties and applications of Mittag-Leffler have been collected, for instance, in references [3, 4]. We also refer to the references [5–7]. For a recent introduction on the Mittag-Leffler functions and its generalizations, the reader may see [6] and [8].

In 2016, Mehrez and Sitnik [9] obtained some Turán type inequalities for Mittag-Leffler functions by considering monotonicity for special ratios of sections for series of Mittag-Leffler functions. Recently, in [10], Yin and Huang also established some Turán type inequalities for the following generalized Mittag-Leffler function via the *p*-gamma function:

1.2 $$ E_{\alpha,\beta,p } (z) = \sum_{n = 0}^{\infty}{ \frac{{z^{n} }}{{\Gamma_{p} ( {\alpha n + \beta} )}},\quad\alpha,\beta,z \in\mathbb{C},p \in(0, \infty),{ \operatorname{Re} } } (\alpha) > 0,{\operatorname{Re} } (\beta) > 0. $$

Motivated by [9, 10], we consider the following generalized Mittag-Leffler function with four parameters:

1.3 $$ E_{\alpha,\beta,p,k } (z) = \sum_{n = 0}^{\infty}{ \frac{{z^{n} }}{{\Gamma_{p,k} ( {\alpha n + \beta} )}},\quad\alpha,\beta ,z \in\mathbb{C},p ,k\in(0, \infty),{ \operatorname{Re} } } (\alpha) > 0,{\operatorname{Re} } (\beta) > 0, $$

where $\Gamma_{p,k}( x )$ is a classical $(p,k)$-gamma function defined by

$$\Gamma_{p,k}( x )=\frac{p!k^{p} (kp)^{\frac{x}{k-1}}}{(x)_{p,k}} $$

and

$$(x)_{p,k}=x(x+k)\cdots\bigl(x+(p-1)k\bigr). $$

It is easily seen that the functions (1.2) and (1.3) are special cases of Wright-Fox functions in the Wright series representation (or multi-index Mittag-Leffler functions) in [11].

It is well known that $\lim _{p \to\infty} \Gamma _{p,k}(x) = \Gamma_{\infty,k}(x)=\Gamma_{k}(x)$, and $\Gamma_{\infty,1}(x)=\Gamma(x)$, where $\Gamma_{k} (x) = \frac{{k!k^{x} }}{{x(x + 1) \cdots(x + p)}} $ and $\Gamma(x) = \int_{0}^{\infty}{t^{x - 1} e^{ - t} }\, dt$, $x > 0$ are *k*-gamma and gamma functions, respectively. These formulas and more properties can be found in [2].

The logarithmic derivative of the $(p,k)$-gamma function

$$\psi_{p,k} (x) = \frac{d}{{dx}}\log\Gamma_{p,k} (x) = \frac {{\Gamma'_{p,k}(x)}}{{\Gamma_{p,k}(x)}} $$

is known as a generalized digamma function. Its derivatives $\psi _{p,k}^{(n)} (x)$ are known as generalized polygamma functions.

Our results read as follows.

### Theorem 1.1

*For* $\alpha,\beta,p,k>0$ *and fixed* $z>0$, *the function* $f:\beta \mapsto\Gamma_{p,k}( \beta)E_{\alpha,\beta,p,k } (z) $ *is strictly log*-*convex on* $(0,\infty)$. *As a result*, *we have the following inequality*:

1.4 $$ {E_{\alpha,\beta+k,p,k }}^{2} (z)< \frac{(\beta+k)(\beta+pk)}{\beta (\beta+(p+1)k)}E_{\alpha,\beta,p,k }(z) E_{\alpha,\beta+2k,p,k }(z). $$

### Corollary 1.1

*For* $\alpha,p,k>0$, $\beta_{2}>\beta_{1}>0$ *and fixed* $z\in(0,\infty)$, *we have*

1.5 $$ \frac{E_{\alpha,\beta_{1}+k,p,k}(z)}{E_{\alpha,\beta _{1},p,k}(z)}< \frac{\beta_{2}(\beta_{1}+pk)}{\beta_{1}(\beta _{2}+pk)}\frac{E_{\alpha,\beta_{2}+k,p,k}(z)}{E_{\alpha,\beta_{2},p,k}(z)}. $$

Putting

1.6 $$ E_{\alpha,\beta,p,k }^{n} (z)=E_{\alpha,\beta,p,k } (z)-\sum _{m = 0}^{n}\frac{z^{m} }{\Gamma_{p,k}(\alpha m + \beta)}= \sum _{m = n+1}^{\infty}\frac{z^{m}}{\Gamma_{p,k}(\alpha m + \beta)}, $$

we obtain the following results.

### Theorem 1.2

*For* $n\in\mathbb{N}$, $\alpha,\beta,p,k,z>0 $, *we have*

1.7 $$ E_{\alpha,\beta,p,k }^{n} (z) E_{\alpha,\beta,p,k }^{n+2} (z)\leq \bigl[ E_{\alpha,\beta,p,k }^{n+1} (z) \bigr]^{2}. $$

### Remark 1.1

For proofs we apply a method introduced and studied in detail in Sitnik and Mehrez (see [9, 12–14]).

## Lemmas

### Lemma 2.1

[12]

*Let* $\{a_{n}\}$ *and* $\{b_{n}\}$ ($n=0,1,2,\ldots$) *be real numbers*, *such that* $b_{n}>0$ *and* $\{\frac{a_{n}}{b_{n}}\}_{n\geq0}$ *is increasing* (*decreasing*). *Then* $\{\frac{a_{0}+a_{1}+\cdots+a_{n}}{b_{0}+b_{1}+\cdots+b_{n}}\}$ *is increasing* (*decreasing*).

### Lemma 2.2

[9]

*Let* $\{a_{n}\}$ *and* $\{b_{n}\}$ ($n=0,1,2,\ldots$) *be real numbers and let the power series* $A(x)=\sum_{n=0}^{\infty}a_{n}x^{n}$ *and* $B(x)=\sum_{n=0}^{\infty}b_{n}x^{n}$ *be convergent if* $|x|< r$. *If* $b_{n}>0$ ($n=0,1,2,\ldots$) *and the sequence* $\{\frac{a_{n}}{b_{n}}\} _{n\geq0}$ *is* (*strictly*) *increasing* (*decreasing*), *then the function* $\frac {A(x)}{B(x)}$ *is also* (*strictly*) *increasing* (*decreasing*) *on* $[0,r)$.

## Proofs of main results

### Proof of Theorem 1.1

Simple computation yields

$$\begin{gathered} \frac{\partial}{\partial\beta} \biggl(\log\frac{\Gamma _{p,k}(\beta)}{\Gamma_{p,k}(\alpha k+\beta)} \biggr) \\\quad=\frac{\Gamma_{p,k}(\alpha k+\beta)}{\Gamma_{p,k}(\beta)}\cdot \frac{\Gamma'_{p,k}(\beta)\Gamma_{p,k}(\alpha k+\beta)-\Gamma _{p,k}(\beta)\Gamma'_{p,k}(\alpha k+\beta)}{(\Gamma_{p,k}(\alpha k+\beta))^{2}} \\\quad=\psi_{p,k}(\beta)-\psi_{p,k}(\alpha k+\beta), \end{gathered}$$

and

$$\frac{\partial^{2}}{\partial\beta^{2}} \biggl(\log\frac{\Gamma _{p,k}(\beta)}{\Gamma_{p,k}(\alpha k+\beta)} \biggr)=\psi '_{p,k}(\beta)-\psi'_{p,k}(\alpha k+ \beta)< 0, $$

where we apply that the function $\psi_{p,k}(x)$ is concave on $\mathbb{R}$. Therefore, we find that the function $\beta\mapsto\frac{\Gamma_{p,k}(\beta)}{\Gamma_{p,k}(\alpha k+\beta)}$ is strictly log-convex on $(0,\infty)$. Using the fact that the sum of log-convex functions is also log-convex, we see that the function *f* is strictly log-convex on $(0,\infty)$.

Due to inequality (1.4), we easily derive

$$\log f \biggl(\frac{\beta+\beta+k}{2}\biggr)< \frac{\log f(\beta)+\log f(\beta+2k)}{2}. $$

That is,

$${E_{\alpha,\beta+k,p,k }}^{2} (z)< \frac{\Gamma_{p,k}(\beta)\Gamma _{p,k}(\beta+2k)}{[\Gamma_{p,k}(\beta+k)]^{2}} E_{\alpha,\beta,p,k } (z)E_{\alpha,\beta+2k,p,k } (z). $$

Using the definition of $\Gamma_{p,k}(x)$, we easily obtain

$$\frac{\Gamma_{p,k}(\beta)\Gamma_{p,k}(\beta+2k)}{[\Gamma _{p,k}(\beta+k)]^{2}}=\frac{\frac{p!k^{p} (kp)^{\frac{\beta }{k-1}}}{(\beta)_{p,k}}\frac{p!k^{p} (kp)^{\frac{\beta +2k}{k-1}}}{(\beta+2k)_{p,k}}}{\frac{p!k^{p} (kp)^{\frac{\beta +k}{k-1}}}{(\beta+k)_{p,k}}}=\frac{(\beta+k)(\beta+pk)}{\beta (\beta+(p+1)k)}, $$

so we have

$${E_{\alpha,\beta+k,p,k }}^{2} (z)< \frac{(\beta+k)(\beta+pk)}{\beta (\beta+(p+1)k)}E_{\alpha,\beta,p,k } (z) E_{\alpha,\beta+2k,p,k } (z). $$

The proof of Theorem 1.1 is complete. □

### Proof of Corollary 1.1

Since the function $f(\beta)$ is strictly log-convex, we see that the function

$$\frac{f(\beta+k)}{f(\beta)}=\frac{\Gamma_{p,k}(\beta+k)E_{\alpha ,\beta+k,p,k } (z)}{\Gamma_{p,k}(\beta)E_{\alpha,\beta,p,k } (z)} $$

is strictly increasing on $(0,\infty)$. By taking $0<\beta_{1}<\beta _{2}$, we have

$$\frac{\Gamma_{p,k}(\beta_{1}+k)E_{\alpha,\beta_{1}+k,p.k } (z)}{\Gamma _{p,k}(\beta_{1})E_{\alpha,\beta_{1},p,k } (z)}< \frac{\Gamma _{p,k}(\beta_{2}+k)E_{\alpha,\beta_{2}+k,p,k } (z)}{\Gamma_{p,k}(\beta _{2})E_{\alpha,\beta_{2},p,k } (z)}. $$

By using the formula

$$\begin{aligned} \frac{\Gamma_{p,k}(\beta_{2}+k)}{\Gamma_{p,k}(\beta_{2})}\cdot\frac {\Gamma_{p,k}(\beta_{1})}{\Gamma_{p,k}(\beta_{1}+k)} &=\frac{\frac{p!k^{p} (kp)^{\frac{\beta_{2}+k}{k-1}}}{(\beta _{2}+k)_{p,k}}}{\frac{p!k^{p} (kp)^{\frac{\beta_{2}}{k-1}}}{(\beta _{2})_{p,k}}}\cdot \frac{\frac{p!k^{p} (kp)^{\frac{\beta_{1}}{k-1}}}{(\beta _{1})_{p,k}}}{\frac{p!k^{p} (kp)^{\frac{\beta_{1}+k}{k-1}}}{(\beta _{1}+k)_{p,k}}} \\&=\frac{(\beta_{2})_{p,k}(\beta_{1}+k)_{p,k}}{(\beta_{1})_{p,k}(\beta_{2}+k)_{p,k}} =\frac{\beta_{2}(\beta_{1}+pk)}{\beta_{1}(\beta_{2}+pk)}, \end{aligned}$$

we complete the proof. □

### Proof of Theorem 1.2

Using the formulas

$$E_{\alpha,\beta,p,k }^{n} (z)=E_{\alpha,\beta,p,k }^{n+1} (z)+ \frac {z^{n+1}}{\Gamma_{p,k}[\alpha(n+1)+\beta]} $$

and

$$E_{\alpha,\beta,p,k }^{n+2} (z)=E_{\alpha,\beta,p,k }^{n+1} (z)- \frac{z^{n+2}}{\Gamma_{p,k}[\alpha(n+2)+\beta]}, $$

we have

$$\begin{gathered} E_{\alpha,\beta,p,k }^{n} (z)E_{\alpha,\beta,p,k }^{n+2} (z)- \bigl[E_{\alpha,\beta,p,k }^{n+1} (z)\bigr]^{2} \\\quad=\biggl[E_{\alpha,\beta,p,k }^{n+1} (z)+\frac{z^{n+1}}{\Gamma _{p,k}[\alpha(n+1)+\beta]}\biggr]\cdot \biggl[E_{\alpha,\beta,p,k }^{n+1} (z)-\frac{z^{n+2}}{\Gamma_{p,k}[\alpha(n+2)+\beta]}\biggr] \\\quad=E_{\alpha,\beta,p,k }^{n+1} (z) \biggl[\frac{z^{n+1}}{\Gamma _{p,k}[\alpha(n+1)+\beta]}- \frac{z^{n+2}}{\Gamma_{p,k}[\alpha (n+2)+\beta]} \biggr]\\ \qquad{}- \frac{z^{2n+3}}{\Gamma_{p,k}[\alpha(n+1)+\beta]\Gamma_{p,k}[\alpha (n+2)+\beta]} \\\quad=\sum_{m=n+3}^{\infty}\frac{z^{n+m+1}}{\Gamma_{p,k}(\alpha m+\beta )\Gamma_{p,k}[\alpha(n+1)+\beta]}\\ \qquad{}-\sum _{m=n+3}^{\infty}\frac {z^{n+m+1}}{\Gamma_{p,k}[\alpha(m-1)+\beta]\Gamma_{p,k}[\alpha (n+2)+\beta]} \\\quad=\sum_{m=n+3}^{\infty}\frac{\Gamma_{p,k}[\alpha(m-1)+\beta]\Gamma _{p,k}[\alpha(n+2)+\beta]-\Gamma_{p,k}(\alpha m+\beta)\Gamma _{p,k}[\alpha(n+1)+\beta]}{\Gamma_{p,k}(\alpha m+\beta)\Gamma _{p,k}[\alpha(n+1)+\beta]\Gamma_{p,k}[\alpha(m-1)+\beta]\Gamma _{p,k}[\alpha(n+2)+\beta]}\\ \qquad{}\times z^{n+m+1}. \end{gathered}$$

Since the function $\Gamma_{p,k}(x)$ is log-convex on $(0,\infty)$, we know that the function $x\mapsto\frac{\Gamma_{p,k}(x+a)}{\Gamma _{p,k}(x)}$ ($a>0$) is increasing on $(0,\infty)$. Thus, with $a=\alpha$, $x=\alpha (n+1)+\beta<\alpha(n+1)+\beta+\alpha(m-(n+2))$ and using Lemma 2.1 and Lemma 2.2, we obtain

$$\frac{\Gamma_{p,k}[\beta+\alpha(n+1)+\alpha]}{\Gamma_{p,k}[\beta +\alpha(n+1)]}\leq\frac{\Gamma_{p,k}[\beta+\alpha(n+1)+\alpha +\alpha(m-(n+2))]}{\Gamma_{p,k}[\beta+\alpha(n+1)+\alpha(m-(n+2))]}. $$

That is,

$$\frac{\Gamma_{p,k}[\alpha(n+2)+\beta]}{\Gamma_{p,k}[\alpha (n+1)+\beta]}\leq\frac{\Gamma_{p,k}(\alpha m+\beta)}{\Gamma _{p,k}[\alpha(m-1)+\beta]}. $$

It follows that

$$E_{\alpha,\beta,p,k }^{n} (z) E_{\alpha,\beta,p,k }^{n+2} (z)- \bigl[ E_{\alpha,\beta,p,k }^{n+1} (z) \bigr]^{2}\geq0. $$

 □

## Conclusions

In this paper, we show several Turán type inequalities for a generalized Mittag-Leffler function with four parameters via the $(p,k)$-gamma function, and we generalize some known results.
